# Farmed cricket performance remains stable over five generations of rearing on a waste-based diet

**DOI:** 10.1093/jee/toag089

**Published:** 2026-04-16

**Authors:** Sophie Y Kasdorf, Susan M Bertram, Heath A MacMillan

**Keywords:** brewery waste, circular bioeconomy, growth, multigenerational, transgenerational effect

## Abstract

Farmed insects like crickets offer a sustainable protein source to feed the growing global population. A benefit of cricket farming is the potential to use waste diets instead of unsustainable, expensive feeds. Brewer’s spent grain is a nutritionally valuable organic waste product that has been used to rear crickets in single-generation studies. However, long-term effects of spent grain-based feeds are unclear, which makes incorporation into commercial feed risky for producers. We reared a farmed cricket *Gryllodes sigillatus* (Orthoptera: Gryllidae) for 5 generations on a high inclusion (75%) spent grain diet. Crickets reared on spent grain were 19% smaller at adulthood than crickets reared on farm feed, resulting in decreased yield (mass of crickets harvested), but were able to reproduce and had high survival rates. Cricket performance remained stable over 5 generations, indicating that spent grain contains adequate nutrition to support long-term cricket production. We also reared crickets on a gradual inclusion spent grain diet that increased from 15% to 75% spent grain over 5 generations. While this “weaning” approach did not improve cricket performance on high-inclusion spent grain diets, crickets on low inclusion diets (15%) displayed about a 40% increase in survival and yield compared to the control. Therefore, inclusion of low amounts of spent grain in cricket feed may not only be beneficial from an environmental and feed cost perspective, but also from a production yield perspective. Our findings are the first to show that spent grain could act as a feed ingredient for long-term rearing of farmed crickets.

## Graphical Abstract

**Graphical Abstract:**
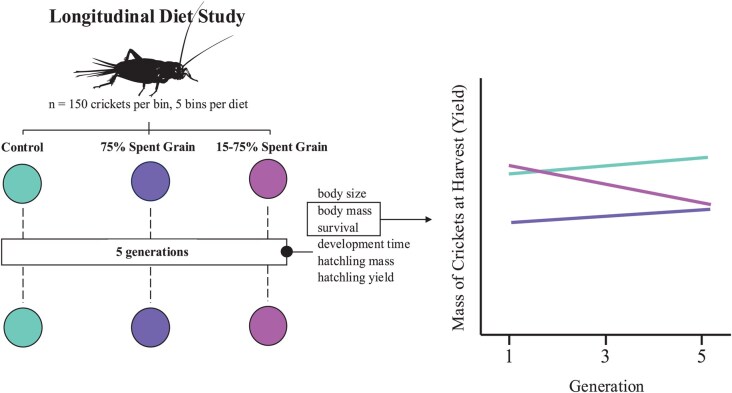

## Introduction

Farmed insects are gaining popularity as a source of sustainable food and feed, leading to a growing market for insect production in North America (Larouche et al. 2023). Production success of the farmed insect industry is heavily dependent on the quantity of insect biomass produced, also known as yield. Yield is influenced by insect life history traits like survival, body mass, development time, and fecundity (Muzzatti et al. 2024, Kong et al. 2025a). Insect life history is shaped by independent and interactive effects of many external factors, including diet (Muzzatti et al. 2024), temperature (Eberle et al. 2022), and rearing density (Mahavidanage et al. 2023). While ideal production conditions would optimize all of these traits, finding trait optima requires close collaboration between industry and academia, connecting fundamental research on insect physiology and ecology with the needs of producers (Tomberlin et al. 2022, Larouche et al. 2023, Kong et al. 2025a).

Feed ingredient choice is a particularly important consideration for insect producers due to a need to balance nutritional value and feed cost. Constraints on nutrient availability in feed relative to insect nutritional requirements lead to a reduction in productivity. For example, insufficient dietary protein may limit nitrogen availability to allocate toward egg production, resulting in lower egg counts (Boggs 2009). Many producers rely on traditional, grain-based feeds similar to those used for vertebrate livestock that have a balanced nutritional profile capable of supporting high rates of insect growth and survival (Lundy and Parrella 2015, Oonincx et al. 2015, Sorjonen et al. 2019). However, traditional feed ingredients like corn, soy, and fishmeal are expensive (Donohue and Cunningham 2009, Macusi et al. 2023), and this is an impediment to the economic viability of the edible insect industry (Halloran et al. 2017, Biteau et al. 2024). In addition, feed crops contribute significantly toward the environmental impact of livestock production, and, combined with pastures, use 40% of arable land on earth (Mottet et al. 2017). Therefore, there is a need to reduce both the economic and environmental costs of insect feed to support industry growth and global sustainability, while maintaining production success.

High-volume organic waste products like brewer’s spent grain are potential alternatives to traditional feed for insect rearing. The brewing industry produces over 36.4 million tonnes of spent grain worldwide each year (Zeko-Pivač et al. 2022), which is composed mainly of spent barley grain containing 15% to 24% protein (Mussatto et al. 2006, Thiago et al. 2014, Rachwał et al. 2020). Therefore, this product is both a valuable source of nutrition and relatively ubiquitous. Multiple farmed insect species have been raised on diets comprised fully or partially of spent grain, including several species of farmed cricket (Oonincx et al. 2015, Orinda et al. 2017, Sorjonen et al. 2019, Jucker et al. 2022, Kasdorf et al. 2025), black soldier flies (Bava et al. 2019, Resconi et al. 2024), and mealworms (van Broekhoven et al. 2015). Replacement of 30% soy with spent grain in cricket feed resulted in a 29% decrease in feed cost per kilogram (Taesuk et al. 2024), demonstrating that spent grain acts as a cost-effective feed ingredient for producers. Recovery of spent grain for commercial insect production also provides a key opportunity to contribute towards the establishment of a circular economy (CE). The CE framework aims to minimize resource consumption and waste output to support sustainable agriculture (Velasco-Muñoz et al. 2021), and the framework is mentioned as a potential benefit of insect farming due to insects’ capacity to convert waste (Madau et al. 2020). However, spent grain inclusion in insect diets can lead to reductions in growth (Lundy and Parrella 2015, Sorjonen et al. 2019, Kasdorf et al. 2025), and the advantages and disadvantages of spent grain diets need to be deeply understood before producers are likely to include this product in their feed.

To date, studies investigating the effects of spent grain inclusion in mass-rearing diets are almost exclusively performed on a single generation of insects. A key knowledge gap is whether or not farmed insects will respond differently to these novel diets over the long term. By omitting measurements of important factors like reproductive success or changes in life history traits (ie performance) over time, these studies cannot reveal multigenerational benefits or costs of waste inclusion. Long-term declines in insect performance may be experienced due to a compounding effect of malnutrition or epigenetic factors. For example, spruce budworm (*Choristoneura fumiferana*) fed a low-quality diet for 4 generations experienced negative impacts on life history, like increased mortality and reduced female reproductive output that accumulated over time (Frago and Bauce 2014). Conversely, black soldier fly larvae (*Hermetia illucens*) reared on a poor diet (wheat bran) displayed an increase in performance traits like larval mass after 12 generations (Gligorescu et al. 2023). As in these examples, a deeper understanding of spent-grain effects on insect life history would assist producers in making informed decisions about the potential risks and benefits of integrating waste-based diets into large-scale commercial farming.

Here, we investigate the impacts of multigenerational rearing of farmed crickets *Gryllodes sigillatus* (Orthoptera: Gryllidae), a popular species for commercial production (Zielińska et al. 2015), on spent-grain-based diets. We maintained crickets for 5 generations on one of 3 experimental diets: a control (existing farm feed), a high inclusion diet (75% spent grain), and a gradual inclusion diet (increased incrementally from 15% to 75% spent grain over the course of the 5 generations). The purpose of the gradual inclusion diet was to investigate a “weaning” approach of gradually introducing a novel diet through multiple intermediary diets that has been used to successfully facilitate the transition of fish to formulated diets in aquaculture (Kubitza and Lovshin 1999, Ljubobratović et al. 2015, Gisbert et al. 2016), although typically in a single generation.

## Materials and Methods

### Diet Treatments

Brewer’s spent grain, composed primarily of barley with wheat and oats, was generously provided by Stray Dog Brewing Company in Ottawa, Ontario, Canada. The spent grain was dried in a drying oven (31574, Precision Scientific Co, Chicago, United States) at 30 °C for approximately 10 d, then ground using a Vevor Commercial Grinding Machine and stored at –20 °C. A pre-mixed farm feed was used as a control. Raw feed components were obtained, dried, and ground from Campbellford Farm Supply in Campbellford, Ontario, Canada. Five experimental diets were created in which the control feed was replaced by mass with spent grain at a proportion of 15%, 30%, 45%, 60%, or 75% (Table 1). Micronutrient supplements (Hog Grower Premix, salt, CaCO_3_, phosphate) used in the farm feed were kept at a consistent proportion in all diets.

**Table 1.** Ingredient composition (g) of diets

Ingredients (g)	Proportion spent grain					
	0% (Control)	15%	30%	45%	60%	75%
**Ground corn**	41.22	35.51	28.71	22.11	15.50	8.90
**soymeal**	39.22	33.04	26.86	20.68	14.51	8.33
**Canola meal**	5.00	4.21	3.42	2.64	1.85	1.06
**Fishmeal**	5.00	4.21	3.42	2.64	1.85	1.06
**Corn gluten meal**	4.09	3.45	2.16	2.16	1.51	0.87
**Spent grain**	N/A	15.00	30.00	45.00	60.00	75.00
**Hog grower vitamin and mineral premix**	1.10	1.10	1.10	1.10	1.10	1.10
**Salt**	0.46	0.46	0.46	0.46	0.46	0.46
**CaCO_3_**	1.61	1.61	1.61	1.61	1.61	1.61
**Phosphate**	1.61	1.61	1.61	1.61	1.61	1.61
**Total mass (g)**	100	100	100	100	100	100

### Rearing

Newly hatched crickets (*Gryllodes sigillatus*) were obtained from a stock population maintained at Carleton University that originated from Entomo Farms in Norwood, Canada. Hatchlings were randomly allocated to 1 of 3 diet treatments: a Control, High Inclusion (75%) Spent Grain, or Gradual Inclusion (15%-75%) Spent Grain. Each diet was provided to crickets in 5 replicate plastic bins (49.5 × 38.1 × 35.2 cm) containing an egg carton for shelter, a plastic vial of water plugged with cotton, and mesh in the lid for aeration (*n* ≈ 150 hatchlings per bin, estimated by mass). Crickets were allowed to feed *ab libitum* and all bins were kept in a greenhouse at 30 °C, approximately 25% humidity and a 14 L:10D photoperiod generated using LEDs for the duration of the experiment.

The experiment was run for 5 generations. Ten days after the first adult was observed in the majority of bins on a treatment, crickets were provided with damp peat moss for oviposition. After 2-d of oviposition, the peat moss was removed to allow egg incubation (30 °C, 25% humidity, 14 L:10D) and hatchling emergence, and adult crickets were harvested (culled). A subset of the hatchlings was returned to the parental bin to restart the cycle (*n* ≈ 150 hatchlings per bin, estimated by mass). Using data from a different project, we confirmed a strong correlation between hatchling number estimated by mass and hatchling number determined by count (*R*^2^ = 0.84; *F* = 65.08; df = 1, 12; *P* < 0.001; *n* = 14).

### Data Collection

**Fig. 1.**
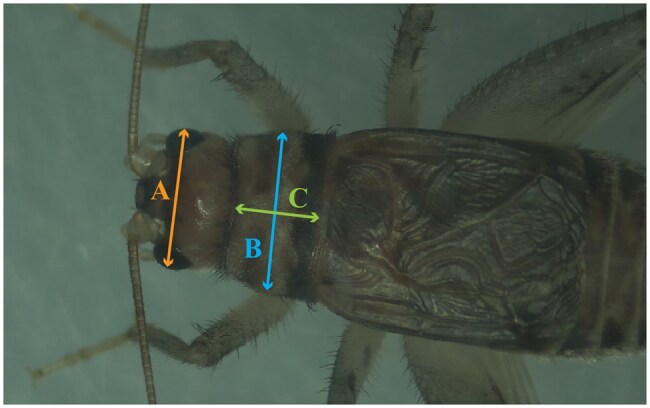
Adult male *G. sigillatus* with labels indicating A) head width, B) pronotum width and C) pronotum length.

To monitor changes in life history traits over time, we quantified cricket adult body mass, adult body size, time to adulthood, survival rate, harvest yield, hatching yield and hatchling mass for each generation. At the end of every generation immediately before harvest, we obtained adult body mass and body size measurements (*n* = 10 crickets per bin, pseudorandomly selected). Mass was obtained using an analytical balance (AB135-S, Mettler Toledo, Columbus, United States), and body size was measured by capturing an image of each cricket using a microscope (Stemi 508, ZEISS, Oberkochen, Germany) with a color camera (Axiocam 208, ZEISS) and ZEN Lite (ZEISS) software (Visanuvimol and Bertram 2011). Head width, pronotum width and pronotum length measurements were obtained from the images using ImageJ v.1.53 software (National Institutes of Health, Bethesda, Maryland, United States) (Fig. 1). Time to first adult emergence (by bin) was recorded as the number of days between the date that hatchlings were added to a bin and the date that adults were first observed. Survival rate (by bin) was quantified by counting the number of living adult crickets in each bin at the end of every generation and dividing it by the starting number (estimated hatchlings by weight). Harvest yield (by bin) was defined as the total mass of crickets produced at the end of every generation, and was quantified by weighing all crickets as a group using an analytical balance (PA224 Pioneer Plus, OHAUS, Parisippany, United States). Hatchling yield (by bin) was defined as the total mass of hatchlings that emerged from peat moss (egg-laying substrate) within 2 d of hatching start, and was quantified by weighing all hatchings as a group using an analytical balance (AB135-S, Mettler Toledo, Columbus, United States). We obtained hatchling body mass measurements (*n* = random subset of 10 hatchlings per bin) at the start of every generation using the same balance.

### Statistical Analyses

All statistical analyses were performed using R v4.2.3 (R Core Team 2024) software. Principal component analyses were used to obtain one value representative of overall body size using three body size measurements (head width, pronotum width, pronotum length) obtained for the adult crickets (Visanuvimol and Bertram 2011). The PC1 size value accounted for 93.4% (eigenvalue = 2.80) of total variance and was positively correlated with head width (loading score = 0.577), pronotum width (loading score = 0.584), and pronotum length (loading score = 0.570), indicating that it is a good representation of cricket size.

To investigate how diet influenced life history traits across generations, we analyzed adult cricket body mass, body size, harvest yield, time to first adult emergence, hatchling mass and hatchling yield using linear mixed effects models fit with the *lme4* package (Bates et al. 2015). Adult and hatchling mass were log_10_ transformed to meet model assumptions. Survival (number of living vs. dead crickets) was analyzed using a generalized linear mixed effects model with a binomial response and a logit link function fit with the *glmer* package (Anderson et al. 2017). Diet, sex, and generation were included as fixed effects for adult body mass and size, while only diet and generation were used for all other measured traits. Generation was centered on the first generation to facilitate interpretation of trends across time. In all models, replicate bin was included as a random effect to account for non-independence of sampling individuals nested within bins (adult body mass and size, hatchling mass) and repeated sampling of bins (ie lineages) across generations. While the random effect of bin accounted for minimal variance in some cases, occasionally approaching 0, it was included in all models to control for the hierarchal and longitudinal study design. Mixed effects model summaries are provided in Supplementary Tables S1 to S7.

Type III ANOVAs with Satterthwaite’s approximation for degrees of freedom were used to test significance of fixed effects of linear mixed effects models, while Type III Wald chi-squared tests were used for the survival model (Table 2). To clarify differences in values at generation 1 (intercept) and trends across generations (slope) for each diet relative to the control, post hoc pairwise comparisons were conducted using estimated marginal means (EMMs) or estimated marginal trends (EMTs) with the *emmeans* package (Lenth 2017). *P*-values were adjusted for multiple comparisons using Bonferroni correction. Intercept and slope for log_10_-transformed values (adult and hatchling mass) were back-transformed for interpretability (Tables 3 and 4).

**Table 2.** Analysis output of ANOVA on linear mixed effects models for quantified life history traits, indicating the significance of fixed effects

Dependent variable	Factor	Df	*x*	*P* value
**Log adult body mass**	Diet	2, 42	*F* = 53.88	**<0.001**
	Generation	1, 726	*F* = 14.96	**<0.001**
	Sex	1, 726	*F* = 360.95	**<0.001**
	Diet × generation	2, 726	*F* = 37.04	**<0.001**
	Diet × sex	2, 726	*F* = 3.38	**0.034**
	Generation × sex	1, 726	*F* = 0.03	0.862
	Diet × generation × sex	2, 726	*F* = 1.42	0.241
**Adult body PC1 size**	Diet	2, 737	*F* = 23.11	**<0.001**
	Generation	1, 737	*F* = 37.82	**<0.001**
	Sex	1, 737	*F* = 351.29	**<0.001**
	Diet × generation	2, 737	*F* = 9.09	**<0.001**
	Diet × sex	2, 737	*F* = 2.36	0.095
	Generation × sex	1, 737	*F* = 1.73	0.189
	Diet × generation × sex	2, 737	*F* = 1.33	0.265
**Survival rate**	Diet	2	χ^2^ = 141.84	**<0.001**
	Generation	1	χ^2^ = 21.64	**<0.001**
	Diet × generation	2	χ^2^ = 124.53	**<0.001**
**Harvest yield**	Diet	2, 54	*F* = 45.62	**<0.001**
	Generation	1, 57	*F* = 29.52	**<0.001**
	Diet × generation	2, 57	*F* = 29.93	**<0.001**
**Time to first adult emergence**	Diet	2, 69	*F* = 2.05	0.136
	Generation	1, 69	*F* = 26.13	**<0.001**
	Diet × generation	2, 69	*F* = 2.86	0.064
**Log hatchling mass**	Diet	2, 744	*F* = 2.66	0.070
	Generation	1, 744	*F* = 42.49	**<0.001**
	Diet × generation	2, 744	*F* = 8.65	**<0.001**
**Hatchling yield**	Diet	2, 69	*F* = 9.69	**<0.001**
	Generation	1, 69	*F *= 40.75	**<0.001**
	Diet × generation	2, 69	*F* = 4.59	**0.013**

Bolded values denote significance (*P* < 0.05).

**Table 3.** Summary table of intercepts and slopes of linear relationships of adult mass and adult body size across generations for diet and sex

Trait	Diet	Sex	*N*	Intercept (units) ± SE	*P*-value	Slope (units/gen) ± SE	*P*-value
**Adult mass (mg)**	Control	F	25	321 ± 9.05	-	1.54 ± 3.36	-
	HISG	F	25	246 ± 6.94	**<0.001**	5.17 ± 2.58	0.823
	GISG	F	25	352 ± 9.94	0.062	–25.8 ± 3.76	**<0.001**
	Control	M	25	203 ± 5.74	-	2.63 ± 2.13	-
	HISG	M	25	178 ± 5.02	**0.003**	–0.153 ± 1.86	1.000
	GISG	M	25	232 ± 6.56	**0.003**	–14.9 ± 2.47	**<0.001**
**Adult body size (PC1)**	Control	F	25	1.687 ± 0.158	-	–0.107 ± 0.064	-
	HISG	F	25	1.041 ± 0.158	**0.012**	–0.049 ± 0.064	1.000
	GISG	F	25	1.866 ± 0.158	1	–0.225 ± 0.064	0.594
	Control	M	25	–1.001 ± 0.158	-	–0.096 ± 0.064	-
	HISG	M	25	–1.483 ± 0.158	0.095	–0.08 ± 0.064	1.000
	GISG	M	25	–0.164 ± 0.158	**<0.001**	–0.413 ± 0.064	**0.002**

Bolded values denote a significant difference from the control group based on EMMs or EMTs (*P* < 0.05).

F, female; GISG, gradual inclusion spent grain; HISG, high inclusion spent grain; M, male.

**Table 4.** Summary table of intercepts and slopes of linear relationships of survival, harvest yield, time to first adult emergence, hatchling mass and hatchling yield across generations for diet

Trait	Diet	*n*	Intercept (units) ± SE	*P*-value	Slope (units/gen) ± SE	*P* value
**Survival (%)**	Control	5	60.4 ± 1.93	-	2.79 ± 0.6	-
	HISG	5	72.49 ± 1.7	**<0.001**	–0.05 ± 0.53	**0.003**
	GISG	5	87.21 ± 1.08	**<0.001**	–3.47 ± 0.42	**<0.001**
**Harvest yield (g)**	Control	5	24.8 ± 1.01	-	0.329 ± 0.399	-
	HISG	5	22.6 ± 1.01	0.369	–0.341 ± 0.399	0.72
	GISG	5	35.3 ± 1.01	**<0.001**	–3.74 ± 0.399	**<0.001**
**Time to first adult (days)**	Control	5	29.6 ± 0.564	-	–0.54 ± 0.23	-
	HISG	5	30.6 ± 0.564	0.646	–1.12 ± 0.23	0.241
	GISG	5	29 ± 0.564	1.000	–0.38 ± 0.23	1.000
**Hatchling mass (mg)**	Control	50	0.968 ± 0.024	-	–0.0683 ± 0.0010	-
	HISG	50	0.893 ± 0.022	0.072	–0.0103 ± 0.0091	**<0.001**
	GISG	50	0.922 ± 0.023	0.523	–0.0301 ± 0.0094	**0.026**
**Hatchling yield (g)**	Control	5	4.47 ± 0.409	-	–0.727 ± 0.167	-
	HISG	5	2.62 ± 0.409	**0.007**	–0.215 ± 0.167	0.103
	GISG	5	5.06 ± 0.409	0.932	–0.903 ± 0.167	1.000

Bolded values denote a significant difference from the control group based on EMMs or EMTs (*P* < 0.05).

GISG, gradual inclusion spent grain, F, Female, HISG, high inclusion spent grain, M, male.

## Results

### Adult Cricket Body Mass and Size

**Fig. 2.**
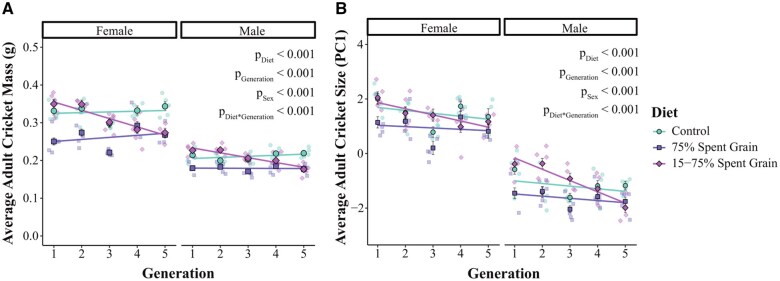
A) Adult mass (g) and B) body size (PC1, first principal component of morphological traits) across 5 generations of crickets reared on a control feed (standard farm feed, circles), a 75% spent grain feed (squares) or a feed that increased incrementally from 15% to 75% spent grain with each generation (diamonds). Each semi-transparent data point represents a replicate cricket colony. Solid points represent mean ± standard error.

Diet treatment significantly impacted adult body mass (*F* = 55.88; df = 2, 42; *P* < 0.001; Fig. 2a) and adult body size (*F* = 23.11; df = 2, 726; *P* < 0.001; Fig. 2b) of *G. sigillatus*. In generation one, crickets reared on the high inclusion spent grain diet were on average 19% lighter (females: 246 ± 7 mg; males: 178 ± 5 mg) than control crickets (females: 321 ± 9 mg, *P* < 0.001; males: 203 ± 6 mg, *P* = 0.008). Female crickets reared on the gradual inclusion spent grain diet weighed similar to control crickets. However, males were about 14% heavier (232 ± 7 mg, *P* = 0.008). Diet and generation also interacted to influence adult body mass (*F* = 37.04; df = 2, 726; *P* < 0.001) and adult body size (*F* = 9.09; df = 2, 737; *P* < 0.001). Crickets reared on the gradual inclusion spent grain diet experienced a progressive decline in body mass across generations as the proportion of spent grain was increased (females: –25.8 ± 3.8 mg gen^−1^, *P* < 0.001; males: –14.9 ± 2.5, *P* < 0.001). In contrast, mass of crickets on the control and the high inclusion spent grain diet remained consistent. Sex explained a significant amount of the variation for both adult body mass (*F* = 360.95; df = 1, 726; *P* < 0.001) and adult body size (*F* = 351.29; df = 1, 737; *P* < 0.001). Generation, a variable used to account for fluctuations due to slight changes in environmental greenhouse conditions between generations, also explained a significant amount of the variation for both adult body mass (*F* = 14.96; df = 1, 726; *P* < 0.001) and adult body size (*F* = 37.82; df = 1, 737; *P* < 0.001), however, it was a weaker effect than the effect of diet or sex. Overall, our findings reveal that while cricket growth was strongly influenced by the proportion of spent grain in the diet, growth remained stable on a high inclusion diet despite a long-term change in diet.

### Survival and Time to Adult Emergence

**Fig. 3.**
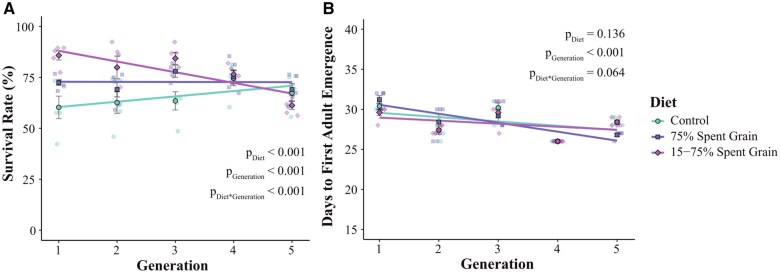
A) Survival rate (%) and B) time to first adult emergence (days) across 5 generations of crickets reared on a control feed (standard farm feed, circles), a 75% spent grain feed (squares) or a feed that increased incrementally from 15% to 75% spent grain with each generation (diamonds). Each semi-transparent data point represents a replicate cricket colony. Solid points represent mean ± standard error.

Diet significantly impacted colony survival rates (χ^2^ = 141.84; df = 2; *P* < 0.001; Fig. 3a). Cricket survival was 20.0% higher on the high inclusion spent grain diet (72.5 ± 1.7%, *P* < 0.001) compared to the control (60.4 ± 1.9%) in the first generation. Survival on the control diet increased over time (2.79 ± 0.60% gen^−1^) while survival on the high inclusion diet remained relatively consistent across generations (–0.05 ± 0.53% gen^−1^; *P* = 0.003). Crickets reared on the gradual inclusion spent grain diet had a 44.4% higher survival rate (87.2 ± 1.1%; *P* < 0.001) in the first generation compared to the control. This positive impact on survival gradually declined over the 5 generations (–3.47 ± 0.42% gen^−1^) as inclusion of the spent grain diet increased. Survival on all diets in the fifth generation was similar. Therefore, spent grain inclusion in the diet led to an increase in survival rate predominantly at low inclusion levels (15%). The time required for the first crickets to emerge to adulthood was similar across diets (*F* = 2.05; df = 1, 69; *P* = 0.136; Fig. 3b); however, the number of days to first adult emergence decreased slightly across generations regardless of diet (*F* = 26.13; df = 1, 69; *P* < 0.001).

### Yield

**Fig. 4.**
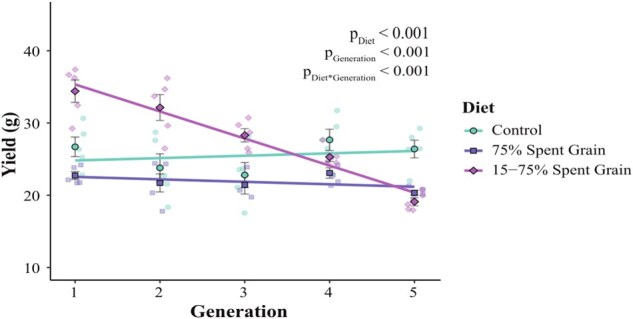
Yield (in g of crickets) across 5 generations of crickets reared on a control feed (standard farm feed, circles), a 75% spent grain feed (squares) or a feed that increased incrementally from 15% to 75% spent grain with each generation (diamonds). Each semi-transparent data point represents a replicate cricket colony. Solid points represent mean ± standard error.

Harvest yield, measured as the total weight of all crickets in the bin at the end of each generation, was significantly impacted by diet treatment (*F* = 45.62; df = 2, 54; *P* < 0.001; Fig. 4). The high inclusion spent grain diet had a harvest yield that was 8.9% less (22.6 ± 1.0 g; *P* = 0.369) than crickets reared on the control diet (24.8 ± 1.0 g). However, this difference was not significant at the experimental scale at which we tested. Yield on the control and the high inclusion spent grain diet remained consistent over time. Diet and generation interacted to influence harvest yield (*F* = 29.05; df = 2, 53; *P* < 0.001); harvest yield on the gradual inclusion spent grain (35.3 ± 1.0 g; *P* < 0.001), was 42.3% higher than the control treatment in the first generation, but gradually declined with successive generations (–3.47 ± 0.40 g gen^−1^) until it was similar to the high inclusion spent grain diet in the fifth generation. Increasing the proportion of spent grain in the cricket diet, therefore, led to decreased harvest yield over successive generations, but never did worse than the introduction of the high-inclusion spent grain diet in a single generation (generation one). Low amounts of spent grain (15%), by contrast, significantly increased yield.

### Hatchling Mass and Hatchling Yield

**Fig. 5.**
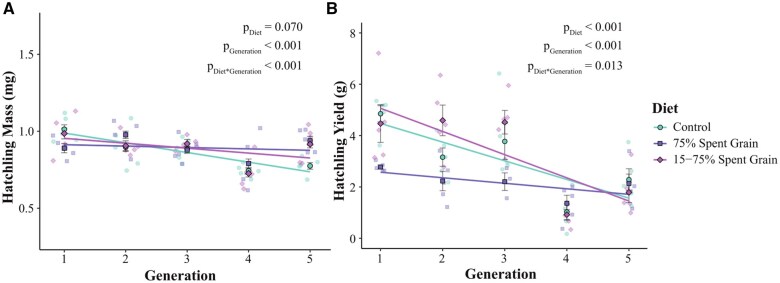
A) Individual hatchling mass (g) and total yield of hatchlings (g) across 5 generations of crickets reared on a control feed (standard farm feed, circles), a 75% spent grain feed (squares) or a feed that increased incrementally from 15% to 75% spent grain with each generation (diamonds). Each semi-transparent data point represents a replicate cricket colony. Solid points represent mean ± standard error.

Diet had a non-significant effect on hatchling mass (*F* = 2.66; df = 2, 744; *P* = 0.070, Fig. 5a), but a stronger effect on hatchling yield (*F* = 9.69; df = 2, 67; *P* < 0.001, Fig. 5b). Hatchling mass displayed a variable trend; hatchlings on the high inclusion spent grain diet tended to be smaller (0.893 ± 0.022 mg; *P* = 0.072) compared to the control (0.968 ± 0.024 mg) in the first generation, but this was not statistically significant. However, hatchling mass on the high inclusion spent grain diet remained relatively consistent (–0.010 ± 0.009 mg/gen; *P* < 0.001), while hatchling mass on the control declined slightly over time (–0.068 ± 0.001 mg/gen). Crickets reared on the high inclusion spent grain diet produced 41.2% fewer hatchlings (2.62 ± 0.41 g; *P* = 0.007) in the first generation compared to the control (4.47 ± 0.41 g). In contrast, the gradual inclusion spent grain (5.06 ± 0.41 g; *P* = 0.932) diets produced a similar mass of hatchlings. However, both the control (–0.727 ± 0.167 g gen^−1^) and the gradual inclusion spent grain diet (–0.903 ± 0.167 g gen^−1^; *P* = 1.000) experienced a decline in hatchling yield. Hatchling yield on the high inclusion spent grain remained more consistent (–0.215 ± 0.167 g gen^−1^; *P* = 0.103); however, the trend did not differ significantly from the control. Therefore, hatchling yield, but not mass, was reduced by a high spent grain content in the diet.

## Discussion

Our results are the first to demonstrate that brewer’s spent grain is a suitable bulk feed ingredient for long-term rearing of commercially produced crickets. We found that adding spent grain to diets has minimal long-term effects on key life history traits important to production of farmed crickets, and that relative performance remains consistent over successive generations. This is promising from a farming perspective, as the consistent performance experienced by crickets raised on a high spent grain diet indicates that cheaper waste-based diets have the potential to work as long-term feed without the risk of colony collapse. By furthering knowledge on the short- and long-term impacts of waste diets on cricket life history traits, our results provide insight into how insects respond to long-term dietary shifts in addition to better informing producers on integration of waste products into commercial insect feed. These findings have important implications for both the edible insect industry and meeting global waste-reduction goals, particularly as low-inclusion spent grain diets have the potential to repurpose organic waste to both reduce feed costs and improve farm productivity.

These results are biologically intriguing, as the lack of multigenerational change provides insight into the relationship between parental conditions and offspring performance. Multiple hypotheses have been proposed on how environmental stressors like suboptimal nutrition in the parental genera­tion may impact offspring fitness through non-genetically transmitted parental effects. For example, a parental stress hypothesis predicts that poor nutrition experienced by the parents may lead to poorer quality offspring (Vijendravarma et al. 2010, Frago and Bauce 2014). Conversely, an adaptive hypothesis predicts that parents reared on a poor diet may be able to better prepare their offspring to better tolerate the same conditions (Mousseau and Fox 1998, Badyaev and Uller 2009, Burgess and Marshall 2014). Both scenarios incorporate transgenerational effects, where the phenotype of the parent influences the phenotype of the offspring (Mousseau and Fox 1998, Shikano et al. 2015). While our experimental design did not directly test for parental effects, our results did not show evidence of a changing response to diet over time, despite phenotypic variation (eg reduced growth) on a high spent grain diet observed in a single generation. These findings support neither an adaptive nor maladaptive transgenerational effects hypothesis, thus raising questions about how insects like crickets remain resilient to nutritional challenges over the long term.

As demonstrated in single-generation studies, crickets are very tolerant of a wide-range of dietary nutritional profiles (Muzzatti et al. 2024, Vadboncoeur et al. 2025). This tolerance may help buffer crickets against transgenerational effects associated with poor parental nutrition. This dietary flexibility is supported by plasticity in behavior and digestive physiology that allows insects to extract essential nutrients from a variety of diet components. For example, insects may behaviorally compensate for dietary imbalance by regulating their feed intake, particularly with respect to protein (Raubenheimer 1992). In our study, crickets raised on a high inclusion (75%) spent grain diet required an increased volume of feed to permit *ab libitum* feeding (Supplementary Fig. S1), supporting the hypothesis that they may be partially adjusting for poor nutrition by consuming more. In addition, physiological mechanisms like regulation of digestive enzyme secretion in the gut can help balance nutrient assimilation (Woodring and Weidlich 2016, Lazarević et al. 2023). Black soldier fly larvae fed a nutritionally unbalanced diet mimicking fruit and vegetable waste displayed differences in digestive enzyme activity in addition to energy reserves and midgut cell morphology (Bonelli et al. 2020). These results were correlated with only moderate impacts of diet on life history (Bonelli et al. 2020), indicating that gut plasticity likely plays a role in compensation for poor nutrition in generalist insects. Future studies exploring digestive markers like enzyme profiles of crickets reared on spent grain or other waste-based diets could help provide insights into the mechanisms of long-term dietary tolerance.

Despite high survival and the completion of successful life cycles across all diets, high inclusion of spent grain reduced adult body mass by about 19%, leading to reductions in overall harvest yield and decreased hatchling output by about 41%. A likely contributor to these changes is the high content of undigestible lignocellulosic material (ie fiber) in spent grain (Aliyu and Bala 2011, Ikram et al. 2017). Fiber is not readily broken down by endogenous enzymes into accessible nutrients and has been linked to nutrient dilution and decreased nutrient absorption in pigs (Bach Knudsen 2001) and poultry (Tejeda and Kim 2021). Development and reproduction are energetically costly processes, and access to dietary protein and digestible carbohydrates in the diet is critical for optimal insect growth (Harrison et al. 2014, Clark et al. 2015, Muzzatti et al. 2024) and fecundity (Rho and Lee 2016, Rapkin et al. 2018, Magara et al. 2019, Gutiérrez et al. 2020). Therefore, a more limited availability of key nutrients to allocate towards growth and reproduction despite compensatory actions likely led to declines in harvest yield for spent grain-fed crickets. Our results align with past (single generation) studies on crickets reared on organic waste-containing diets. For example, *Acheta domesticus* reared on diets in which soy was replaced with spent grain (∼41% of total diet) were slightly smaller than crickets fed a control feed (Sorjonen et al. 2019). Similarly, *Acheta domesticus* reared on mixed waste diets grew smaller than those reared on a control diet, which was linked to a lower nitrogen (protein) to fiber ratio (Lundy and Parrella 2015). While reproductive metrics are less explored in studies on waste diets for farmed crickets, poor nutrition is often linked to decreased maternal investment in insects. For example, crickets (*Scapsipedus icipe*) raised on balanced diets had higher lifetime fecundity than crickets raised on protein-poor diets (Magara et al. 2019). As hatchlings produced on the high spent grain diet were viable and showed no evidence of increased mortality, females may have been prioritizing offspring fitness over offspring number to compensate for limited nutrition. Adjusting investment per offspring to produce fewer, better provisioned offspring is a known life history strategy (Smith and Fretwell 1974). Egg size is positively correlated to fitness in species lacking maternal care (Azevedo et al. 1997), and increases in egg size produced by insects reared in poor diets, despite reduced body size has been documented in species like *Drosophila* (Vijendravarma et al. 2010). This prioritization could potentially explain why crickets produced larger hatchlings on the high inclusion (75%) spent grain diet relative to the control in later generations. Taken together, our findings suggest that while crickets can tolerate high-spent grain diets, nutritional limitations, likely related to fiber content, constrain growth and reproductive output. In particular, the large impact on hatchling output indicates that high inclusion spent grain diets may be better purposed as feed for production animals, rather than feed for breeding stock.

In the event that adaptive transgenerational effects were observed, leading to increased cricket performance on the high inclusion (75%) spent grain diet in the fifth generation compared to the first, we included a gradual inclusion diet to evaluate whether the initial negative impacts could be mitigated using a “weaning” approach. However, cricket growth on this diet linearly decreased with increasing proportions of spent grain over the course of the 5 generations, further supporting the idea that spent grain inclusion influences cricket performance, but that this relationship is not being strongly impacted by parental diet through transgenerational effects. Interestingly, the lower inclusion (15% to 30%) diets in generations one and 2 led up to about a 40% increase in survival and yield. It may be the case that while high levels of dietary fiber lead to nutrient dilution, lower levels increase performance through beneficial actions like promotion of healthy gut microbial communities (Engel and Moran 2013, Douglas 2015). These results suggest that spent grain is a valuable feed ingredient that has the capacity to both reduce costs and increase productivity of cricket farming when used in moderation, reinforcing the role of crickets in food waste reduction and circular bioeconomy initiatives.

Beyond diet, unintentional variations in environmental conditions influenced cricket life history. A reduction in body mass was observed on all 3 diets in the third generation, as well as a reduction in control hatchling yield in the fourth and fifth generations. Although greenhouse conditions were relatively stable throughout the 10-mo experiment, slight fluctuations were accounted for in all models as the “Generation” factor. This factor explained a significant amount of variation for most measured traits, including the control. In the third generation, a relatively modest drop in temperature from a daily average of approximately 30.5 °C to 27 °C was experienced by crickets for roughly 10 d due to a heating system malfunction (Supplementary Fig. S2). Environmental factors like temperature, humidity, and light cycles are known to strongly impact insect life history traits (Ratte 1984, Johnsen et al. 2021), and may also interact with diet (Kingsolver and Woods 1998, Clissold et al. 2013, Kutz et al. 2019). In *Gryllodes sigillatus* crickets, rearing temperatures between 30 °C and 36 °C are considered optimal, with lower temperatures negatively impacting performance (Kong et al. 2025b). Therefore, the fluctuations in insect growth and fecundity observed are likely tied to sub-optimal temperatures during development. Interestingly, the impacts on fecundity were not tied directly to the generation exposed to the temperature drop (generation three), but persisted in the control group into generations 4 and 5. Transgenerational effects of thermal stress in insects remain poorly understood, but both low and high temperature shock have been shown to induce effects on fecundity that last for 2 generations in fall armyworm (Reshma et al. 2023). This suggests that even short-term temperature stressors have profound consequences for insect performance in the long-term, highlighting the need for studies investigating transgenerational plasticity of insects in response to temperature.

Overall, the results of our study demonstrate the capacity for crickets to survive on diets containing spent grain for multiple generations, indicating that spent grain is a promising ingredient for inclusion in cricket feed that could be used as a cost-effective alternative to traditional feed components. While high proportions of spent grain in the diet are nutritionally challenging for crickets and lead to reduced growth compared to a control, crickets demonstrate a remarkable ability to survive and grow on waste-based diets for prolonged periods of time without compounding adverse effects. Replacement of 75% of traditional feed with spent grain only led to about a 10% drop in yield relative to the control, while replacement of lower proportions (15% to 30%) led to up to a 42% increase in yield. This indicates that, in addition to being cost-effective, partial inclusion of spent grain into farm feeds may even increase yield while decreasing production cost.

## Supplementary Material

toag089_Supplementary_Data

## References

[toag089-B1] Aliyu S , Bala M. 2011. Brewer’s spent grain: a review of its potentials and applications. Afr. J. Biotechnol. 10:324–331.

[toag089-B2] Anderson DL , McClure CJW, Franke A. 2017. An introduction to survival analysis using generalized linear mixed models. In: Applied raptor ecology: essentials from gyrfalcon research. The Peregrine Fund. p. 127–146. 10.4080/are.2017/007

[toag089-B3] Azevedo RBR , French V, Partridge L. 1997. Life‐history consequences of egg size in *Drosophila melanogaster*. Am. Nat. 150:250–282. 10.1086/286065 18811284

[toag089-B4] Bach Knudsen KE. 2001. The nutritional significance of “dietary fibre” analysis. Anim. Feed Sci. Technol. 90:3–20. 10.1016/S0377-8401(01)00193-6

[toag089-B5] Badyaev AV , Uller T. 2009. Parental effects in ecology and evolution: mechanisms, processes and implications. Philos. Trans. R Soc. Lond. B Biol. Sci. 364:1169–1177. 10.1098/rstb.2008.0302 19324619 PMC2666689

[toag089-B6] Bates D , Mächler M, Bolker B, et al 2015. Fitting linear mixed-effects models using lme4. J. Stat. Soft. 67:1–48. 10.18637/jss.v067.i01

[toag089-B7] Bava L , Jucker C, Gislon G, et al 2019. Rearing of *Hermetia illucens* on different organic by-products: influence on growth, waste weduction, and environmental impact. Animals 9:289. 10.3390/ani9060289 31146401 PMC6617253

[toag089-B8] Biteau C , Bry-Chevalier T, Crummett D, et al 2024. Insect-based livestock feeds are unlikely to become economically viable in the near future. Food Humanity 3:100383. 10.1016/j.foohum.2024.100383

[toag089-B9] Boggs CL. 2009. Understanding insect life histories and senescence through a resource allocation lens. Funct. Ecol. 23:27–37. 10.1111/j.1365-2435.2009.01527.x

[toag089-B10] Bonelli M , Bruno D, Brilli M, et al 2020. Black soldier fly larvae adapt to different food substrates through morphological and functional responses of the midgut. Int. J. Mol. Sci. 21:4955. 10.3390/ijms21144955 32668813 PMC7404193

[toag089-B11] van Broekhoven S , Oonincx DGAB, van Huis A, et al 2015. Growth performance and feed conversion efficiency of three edible mealworm species (Coleoptera: Tenebrionidae) on diets composed of organic by-products. J. Insect Physiol. 73:1–10. 10.1016/j.jinsphys.2014.12.005 25576652

[toag089-B12] Burgess SC , Marshall DJ. 2014. Adaptive parental effects: the importance of estimating environmental predictability and offspring fitness appropriately. Oikos 123:769–776. 10.1111/oik.01235

[toag089-B13] Clark RM , Zera AJ, Behmer ST. 2015. Nutritional physiology of life-history trade-offs: how food protein–carbohydrate content influences life-history traits in the wing-polymorphic cricket *Gryllus firmus*. J. Exp. Biol. 218:298–308. 10.1242/jeb.112888 25524979

[toag089-B14] Clissold FJ , Coggan N, Simpson SJ. 2013. Insect herbivores can choose microclimates to achieve nutritional homeostasis. J. Exp. Biol. 216:2089–2096. 10.1242/jeb.078782 23430995

[toag089-B15] Donohue M , Cunningham DL. 2009. Effects of grain and oilseed prices on the costs of US poultry production. J. Appl. Poult. Res. 18:325–337. 10.3382/japr.2008-00134

[toag089-B16] Douglas AE. 2015. Multiorganismal insects: diversity and function of resident microorganisms. Annu. Rev. Entomol. 60:17–34. 10.1146/annurev-ento-010814-020822 25341109 PMC4465791

[toag089-B17] Eberle S , Schaden L-M, Tintner J, et al 2022. Effect of temperature and photoperiod on development, survival, and growth rate of mealworms, *Tenebrio molitor*. Insects 13:321. 10.3390/insects13040321 35447763 PMC9029539

[toag089-B18] Engel P , Moran NA. 2013. The gut microbiota of insects–diversity in structure and function. FEMS Microbiol. Rev. 37:699–735. 10.1111/1574-6976.12025 23692388

[toag089-B19] Frago E , Bauce É. 2014. Life-history consequences of chronic nutritional stress in an outbreaking insect defoliator. PLoS One. 9:e88039. 10.1371/journal.pone.0088039 24505368 PMC3914887

[toag089-B20] Gisbert E , Mozanzadeh MT, Kotzamanis Y, et al 2016. Weaning wild flathead grey mullet (*Mugil cephalus*) fry with diets with different levels of fish meal substitution. Aquaculture 462:92–100. 10.1016/j.aquaculture.2016.04.035

[toag089-B21] Gligorescu A , Chen L, Jensen K, et al 2023. Rapid evolutionary adaptation to diet composition in the black soldier fly (*Hermetia illucens*). Insects 14:821. 10.3390/insects14100821 37887833 PMC10607891

[toag089-B22] Gutiérrez Y , Fresch M, Ott D, et al 2020. Diet composition and social environment determine food consumption, phenotype and fecundity in an omnivorous insect. R Soc. Open Sci. 7:200100. 10.1098/rsos.200100 32431901 PMC7211883

[toag089-B23] Halloran A , Hanboonsong Y, Roos N, et al 2017. Life cycle assessment of cricket farming in north-eastern Thailand. J. Clean. Prod. 156:83–94. 10.1016/j.jclepro.2017.04.017

[toag089-B24] Harrison SJ , Raubenheimer D, Simpson SJ, et al 2014. Towards a synthesis of frameworks in nutritional ecology: interacting effects of protein, carbohydrate and phosphorus on field cricket fitness. Proc. R Soc. B 281:20140539. 10.1098/rspb.2014.0539 PMC4150310 25143029

[toag089-B25] Ikram S , Huang L, Zhang H, et al 2017. Composition and nutrient value proposition of brewers spent grain. J. Food Sci. 82:2232–2242. 10.1111/1750-3841.13794 28833108

[toag089-B26] Johnsen NS , Andersen JL, Offenberg J. 2021. The effect of relative humidity on the survival and growth rate of the yellow mealworm larvae (*Tenebrio molitor*, Linnaeus 1758). JIFF. 7:311–318. 10.3920/JIFF2020.0068

[toag089-B27] Jucker C , Belluco S, Oddon SB, et al 2022. Impact of some local organic by-products on *Acheta domesticus* growth and meal production. JIFF. 8:631–640. 10.3920/JIFF2021.0121

[toag089-B28] Kasdorf SY , Muzzatti MJ, Haider F, et al 2025. Brewery waste as a sustainable protein source for the banded cricket (*Gryllodes sigillatus*). J. Insects Food Feed 11:1417–1429. 10.1163/23524588-00001368

[toag089-B29] Kingsolver JG , Woods HA. 1998. Interactions of temperature and dietary protein concentration in growth and feeding of *Manduca sexta* caterpillars. Physiol. Entomol. 23:354–359. 10.1046/j.1365-3032.1998.234105.x

[toag089-B30] Kong JD , Ritchie MW, Vadboncoeur É, et al 2025a. Growth, development, and life history of a mass-reared edible insect, *Gryllodes sigillatus* (Orthoptera: Gryllidae). J. Econ. Entomol. 118:1093–1103. 10.1093/jee/toaf073. 40251933 PMC12167847

[toag089-B31] Kong JD , Vadboncoeur É, Bertram SM, et al 2025b. Temperature-dependence of life history in an edible cricket: implications for optimising mass-rearing. Curr. Res. Insect Sci. 7:100109. 10.1016/j.cris.2025.100109 40129661 PMC11931298

[toag089-B32] Kubitza F , Lovshin LL. 1999. Formulated diets, feeding strategies, and cannibalism control during intensive culture of juvenile carnivorous fishes. Rev. Fish. Sci. 7:1–22. 10.1080/10641269991319171

[toag089-B33] Kutz TC , Sgrò CM, Mirth CK. 2019. Interacting with change: diet mediates how larvae respond to their thermal environment. Funct. Ecol. 33:1940–1951. 10.1111/1365-2435.13414

[toag089-B34] Larouche J , Campbell B, Hénault-Éthier L, et al 2023. The edible insect sector in Canada and the United States. Anim. Front. 13:16–25. 10.1093/af/vfad047 PMC10425141 37583805

[toag089-B35] Lazarević J , Milanović S, Šešlija Jovanović D, et al 2023. Temperature- and diet-induced plasticity of growth and digestive enzymes activity in spongy moth larvae. Biomolecules 13:821. 10.3390/biom13050821 37238690 PMC10216847

[toag089-B36] Lenth RV. 2017. emmeans: estimated marginal means, aka least-squares means. 1.11.2-8. 10.32614/CRAN.package.emmeans

[toag089-B37] Ljubobratović U , Kucska B, Feledi T, et al 2015. Effect of weaning strategies on growth and survival of pikeperch, *Sander lucioperca*, larvae. Tukr. J. Fish. Aquat. Sci. 15:325–331.

[toag089-B38] Lundy ME , Parrella MP. 2015. Crickets are not a free lunch: protein capture from scalable organic side-streams via high-density populations of *Acheta domesticus*. PLoS One. 10:e0118785. 10.1371/journal.pone.0118785 25875026 PMC4398359

[toag089-B39] Macusi ED , Cayacay MA, Borazon EQ, et al 2023. Protein fishmeal replacement in aquaculture: a systematic review and implications on growth and adoption viability. Sustainability 15:12500. 10.3390/su151612500

[toag089-B40] Madau FA , Arru B, Furesi R, et al 2020. Insect farming for feed and food production from a circular business model perspective. Sustainability 12:5418. 10.3390/su12135418

[toag089-B41] Magara HJO , Tanga CM, Ayieko MA, et al 2019. Performance of newly described native edible cricket *Scapsipedus icipe* (Orthoptera: Gryllidae) on various diets of relevance for farming. J. Econ. Entomol. 112:653–664. 10.1093/jee/toy397 30657915

[toag089-B42] Mahavidanage S , Fuciarelli TM, Li X, et al 2023. The effects of rearing density on growth, survival, and starvation resistance of the house cricket *Acheta domesticus*. JOR. 32:25–31. 10.3897/jor.32.86496

[toag089-B43] Mottet A , de Haan C, Falcucci A, et al 2017. Livestock: on our plates or eating at our table? A new analysis of the feed/food debate. Global Food Secur. 14:1–8. 10.1016/j.gfs.2017.01.001

[toag089-B44] Mousseau TA , Fox CW. 1998. The adaptive significance of maternal effects. Trends Ecol. Evol. 13:403–407. 10.1016/S0169-5347(98)01472-4 21238360

[toag089-B45] Mussatto SI , Dragone G, Roberto IC. 2006. Brewers’ spent grain: generation, characteristics and potential applications. J. Cereal Sci. 43:1–14. 10.1016/j.jcs.2005.06.001

[toag089-B46] Muzzatti MJ , Harrison SJ, McColville ER, et al 2024. Applying nutritional ecology to optimize diets of crickets raised for food and feed. R Soc. Open Sci. 11:241710. 10.1098/rsos.241710 39635150 PMC11614541

[toag089-B47] Oonincx DGAB , van BS, van HA, et al 2015. Feed conversion, survival and development, and composition of four insect species on diets composed of food by-products. PLoS One. 10:e0144601. 10.1371/journal.pone.0144601 26699129 PMC4689427

[toag089-B48] Orinda MA , Mosi RO, Ayieko MA, et al 2017. Growth performance of common house cricket (*Acheta domesticus*) and field cricket (*Gryllus bimaculatus*) crickets fed on agro-byproducts. J. Entomol. Zool. Stud. 5:1664–1668.

[toag089-B49] Rachwał K , Waśko A, Gustaw K, et al 2020. Utilization of brewery wastes in food industry. PeerJ. 8: e9427. 10.7717/peerj.9427 32742775 PMC7367049

[toag089-B50] Rapkin J , Jensen K, Archer CR, et al 2018. The geometry of nutrient space–based life-history trade-offs: sex-specific effects of macronutrient intake on the trade-off between encapsulation ability and reproductive effort in decorated crickets. Am. Nat. 191:452–474. 10.1086/696147 29570407

[toag089-B51] Ratte HT. 1984. Temperature and insect development. In: Hoffmann KH, editor. Environmental physiology and biochemistry of insects. Springer Berlin Heidelberg. p. 33–66. 10.1007/978-3-642-70020-0_2

[toag089-B52] Raubenheimer D. 1992. Tannic acid, protein, and digestible carbohydrate: dietary imbalance and nutritional compensation in locusts. Ecology 73:1012–1027. 10.2307/1940176

[toag089-B53] Resconi A , Bellezza Oddon S, Ferrocino I, et al 2024. Effects of brewery by-products on growth performance, bioconversion efficiency, nutritional profile, and microbiota and mycobiota of black soldier fly larvae. Animal 18:101288. 10.1016/j.animal.2024.101288 39226779

[toag089-B54] Reshma R , Sagar D, Subramanian S, et al 2023. Transgenerational effects of thermal stress on reproductive physiology of fall armyworm, *Spodoptera frugiperda*. J. Pest Sci. 96:1465–1481. 10.1007/s10340-023-01660-2

[toag089-B55] Rho MS , Lee KP. 2016. Balanced intake of protein and carbohydrate maximizes lifetime reproductive success in the mealworm beetle, *Tenebrio molitor* (Coleoptera: Tenebrionidae). J. Insect Physiol. 91-92:93–99. 10.1016/j.jinsphys.2016.07.002 27405009

[toag089-B56] Shikano I , Oak MC, Halpert-Scanderbeg O, et al 2015. Trade-offs between transgenerational transfer of nutritional stress tolerance and immune priming. Funct. Ecol. 29:1156–1164. 10.1111/1365-2435.12422

[toag089-B57] Smith CC , Fretwell SD. 1974. The optimal balance between size and number of offspring. Am. Nat. 108:499–506.

[toag089-B58] R Core Team. 2024. R: a language and environment for statistical computing [Software]. https://www.R-project.org/.

[toag089-B59] Sorjonen JM , Valtonen A, Hirvisalo E, et al 2019. The plant-based by-product diets for the mass-rearing of *Acheta domesticus* and *Gryllus bimaculatus*. PLoS One. 14:e0218830. 10.1371/journal.pone.0218830 31246993 PMC6597079

[toag089-B60] Taesuk N , Upan J, Siriamornpun S, et al 2024. Utilising brewer’s spent grain as a cost-effective feed formula for sustainable house cricket (*Acheta domesticus*) rearing. J. Insects Food Feed 11:987–999. 10.1163/23524588-00001358

[toag089-B61] Tejeda OJ , Kim WK. 2021. Role of dietary fiber in poultry nutrition. Animals 11:461. 10.3390/ani11020461 33572459 PMC7916228

[toag089-B62] Thiago RDSM , Pedro PMDM, Eliana FCS. 2014. Solid wastes in brewing process: a review. J. Brew. Distill. 5:1–9. 10.5897/JBD2014.0043

[toag089-B63] Tomberlin JK , Picard CJ, Jordan HR, et al 2022. Government and industry investment plays crucial role in further establishment, evolution, and diversification of insect agriculture: a case example from the United States. JIFF. 8:109–111. 10.3920/JIFF2022.x001

[toag089-B64] Vadboncoeur É , Deschamps M-H, Bertram SM, et al 2025. Temperature outweighs diet in shaping developmental performance in two cricket species via growth delays and physiological limits. J. Exp. Biol. 228. 10.1242/jeb.251472 PMC12752487 41200745

[toag089-B65] Velasco-Muñoz JF , Mendoza JMF, Aznar-Sánchez JA, et al 2021. Circular economy implementation in the agricultural sector: definition, strategies and indicators. Resour. Conserv. Recycl. 170:105618. 10.1016/j.resconrec.2021.105618

[toag089-B66] Vijendravarma RK , Narasimha S, Kawecki TJ. 2010. Effects of parental larval diet on egg size and offspring traits in *Drosophila*. Biol. Lett. 6:238–241. 10.1098/rsbl.2009.0754 19875510 PMC2865044

[toag089-B67] Visanuvimol L , Bertram SM. 2011. How dietary phosphorus availability during development influences condition and life history traits of the cricket, *Acheta domesticas*. J. Insect Sci. 11:1–17. 10.1673/031.011.6301 21864157 PMC3281447

[toag089-B68] Woodring J , Weidlich S. 2016. The secretion of digestive enzymes and caecal size are determined by dietary protein in the cricket *Gryllus bimaculatus*. Arch. Insect Biochem. Physiol. 93:121–128. 10.1002/arch.21346 27447828

[toag089-B69] Zeko-Pivač A , Tišma M, Žnidaršič-Plazl P, et al 2022. The potential of brewer’s spent grain in the circular bioeconomy: state of the art and future perspectives. Front. Bioeng. Biotechnol. 10:870744. https://www.frontiersin.org/articles/10.3389/fbioe.2022.870744. 35782493 10.3389/fbioe.2022.870744 PMC9247607

[toag089-B70] Zielińska E , Baraniak B, Karaś M, et al 2015. Selected species of edible insects as a source of nutrient composition. Food Res. Int. 77:460–466. 10.1016/j.foodres.2015.09.008

