# Supplementary material for: Farmed cricket performance remains stable over five generations of rearing on a waste-based diet
Source: J Econ Entomol. 2026 Apr 16;119(3):1688–98. doi: 10.1093/jee/toag089 (PMC13268526; doi:10.1093/jee/toag089)
Supplement: toag089_Supplementary_Data [file toag089_supplementary_data.zip › toag089_Supplementary_Data/Data_File_Metadata.docx]

Metadata

**Farmed cricket performance remains stable over 5 generations**

**of rearing on a waste-based diet**

Sophie Y. Kasdorf, Susan M. Bertram, Heath A. MacMillan

The “Weekly_Mass.csv” data file contains weekly mass, instar and sex data from the multigenerational rearing experiment of crickets (*Gryllodes sigillatus*) placed on three diet treatments (a control, a high spent grain inclusion treatment and a gradual spent grain inclusion treatment). Crickets were maintained in a greenhouse at Carleton University, ON, Canada at 30°C, approximately 25% relative humidity and a 14L:10D photoperiod generated with LEDs. Experiment start date was 2023-12-01 (YYYY-MM-DD) and experiment end date was 2024-08-11 (YYYY-MM-DD).

| ***Column*** | ***Content*** |
| --- | --- |
| *Date* | The data collection date (YYYY-MM-DD). |
| *Day_of_Generation* | The day of data collection relative to the generation start date. |
| *Generation* | Generation number (1-6). |
| *Diet* | Diet treatments (*Control* – standard farm feed; *SG_15* – 15% spent grain inclusion diet; *SG_30* – 30% spent grain inclusion diet; *SG_45* – 45% spent grain inclusion diet; *SG_60* – 60% spent grain inclusion diet; *SG_75* – 75% spent grain inclusion diet; *SG_75_V* – 75% spent grain inclusion diet for gradual inclusion treatment group). |
| *Bin* | Bin number (1-15). |
| *Cricket* | Number (1-10) associated with each of the ten randomly selected crickets. Note that the numbers are **not** ID’s associated with a particular cricket. |
| *Mass* | Mass of the cricket in g. Collected with an AB135-S analytical balance (Mettler Toledo, Columbus, USA) to a decimal of 0.00001. |
| *Instar* | Instar of the cricket. |
| *Sex* | Sex of the cricket (only determined post-adulthood). F – Female, M – Male. |

The “Body_Photograph_Measurements.csv” data file contains body size data from the multigenerational rearing experiment of crickets (*Gryllodes sigillatus*) placed on three diet treatments (a control, and two spent grain inclusion diets). Crickets were maintained in a greenhouse at Carleton University, ON, Canada at 30°C, approximately 25% relative humidity and a 14L:10D photoperiod generated with LEDs. Experiment start date was 2023-12-01 (YYYY-MM-DD) and experiment end date was 2024-08-11 (YYYY-MM-DD).

| ***Column*** | ***Content*** |
| --- | --- |
| *Date* | The data collection date (YYYY-MM-DD). |
| *Generation* | Generation number (1-5). |
| *Diet* | Diet treatments (*Control* – standard farm feed; *SG_15* – 15% spent grain inclusion diet; *SG_30* – 30% spent grain inclusion diet; *SG_45* – 45% spent grain inclusion diet; *SG_60* – 60% spent grain inclusion diet; *SG_75* – 75% spent grain inclusion diet). |
| *Bin* | Bin number (1-15). |
| *Cricket* | Number (1-10) associated with each of the ten randomly selected crickets. Note that the numbers are **not** ID’s associated with a particular cricket. |
| *Head_Width_mm* | Cricket head width measurement in mm obtained from a photograph captured using a Stemi 508 microscope (ZEISS, Oberkochen, Germany) and ZEN Lite (ZEISS, Oberkochen, Germany) software. Measurement was obtained from the photograph using ImageJ v.1.53 software (National Institutes of Health, Bethesda, MD, U.S.A.). |
| *Thorax_Width_mm* | Cricket thorax width measurement in mm obtained from a photograph captured using a Stemi 508 microscope (ZEISS, Oberkochen, Germany) and ZEN Lite (ZEISS, Oberkochen, Germany) software. Measurement was obtained from the photograph using ImageJ v.1.53 software (National Institutes of Health, Bethesda, MD, U.S.A.). |
| *Thorax_Length_mm* | Cricket thorax length measurement in mm obtained from a photograph captured using a Stemi 508 microscope (ZEISS, Oberkochen, Germany) and ZEN Lite (ZEISS, Oberkochen, Germany) software. Measurement was obtained from the photograph using ImageJ v.1.53 software (National Institutes of Health, Bethesda, MD, U.S.A.). |
| *Sex* | Sex of the cricket. |

*note that thorax refers to pronotum and is referred to as such in the manuscript

The “Hatchlings_Harvest_Mass.csv” data file contains yield data from the multigenerational rearing experiment of crickets (*Gryllodes sigillatus*) placed on three diet treatments (a control, and two spent grain inclusion diets). Crickets were maintained in a greenhouse at Carleton University, ON, Canada at 30°C, approximately 25% relative humidity and a 14L:10D photoperiod generated with LEDs. Experiment start date was 2023-12-01 (YYYY-MM-DD) and experiment end date was 2024-08-11 (YYYY-MM-DD).

| ***Column*** | ***Content*** |
| --- | --- |
| *Generation* | Generation number (1-5). |
| *Diet* | Diet treatments (*Control* – standard farm feed; *SG_15* – 15% spent grain inclusion diet; *SG_30* – 30% spent grain inclusion diet; *SG_45* – 45% spent grain inclusion diet; *SG_60* – 60% spent grain inclusion diet; *SG_75* – 75% spent grain inclusion diet). |
| *Bin* | Bin number (1-15). |
| *Mass_Hatchlings* | Total mass of hatchlings in g added to each bin at the start of the generation. Collected with an AB135-S analytical balance (Mettler Toledo, Columbus, USA) to a decimal of 0.00001. |
| *Avg_Hatchling_Mass* | The average hatchling mass (usually by diet) obtained from the ‘Weekly_Mass’ dataset. Used to calculate the total mass of hatchlings needed to obtain 150 hatchlings. |
| *Mass_Harvest_Container* | The mass of the container used to weigh adult crickets collected from each bin at the generation end. Collected with a PA224 Pioneer^Tm^ Plus analytical balance (OHAUS, Parisippany, USA) to a decimal of $\pm$0.0001. |
| *Mass_Harvest* | The combined mass of the container used to weigh crickets AND the crickets collected from each bin at the generation end. Collected with a PA224 Pioneer^Tm^ Plus analytical balance (OHAUS, Parisippany, USA) to a decimal of 0.0001. |
| *End_Females_Count* | The number of adult female crickets harvested at the end of the generation (manually counted). |
| *End_Males_Count* | The number of adult male crickets harvested at the end of the generation (manually counted). |
| *End_Juveniles_Count* | The number of adult female crickets harvested at the end of the generation (manually counted). |
| *Theoretical_Cricket_No* | The theoretical number of total crickets in a bin (excluding six crickets removed for gut dissections or whole-body samples). |
| *Hatchling_Yield* | The mass in g of the hatchlings obtained at the end of a 48-hour hatching period collected with an AB135-S analytical balance (Mettler Toledo, Columbus, USA) to a decimal of 0.00001. |

*Note that bins 9 & 10 were not included in survival analysis for generation three only due to loss of some crickets during harvest.

The “Generation_Duration.csv” data file generation duration data from the multigenerational rearing experiment of crickets (*Gryllodes sigillatus*) placed on three diet treatments (a control, and two spent grain inclusion diets). Crickets were maintained in a greenhouse at Carleton University, ON, Canada at 30°C, approximately 25% relative humidity and a 14L:10D photoperiod generated with LEDs. Experiment start date was 2023-12-01 (YYYY-MM-DD) and experiment end date was 2024-08-11 (YYYY-MM-DD).

| ***Column*** | ***Content*** |
| --- | --- |
| *Generation* | Generation number (1-5). |
| *Diet* | Diet treatments (*Control* – standard farm feed; *SG_15* – 15% spent grain inclusion diet; *SG_30* – 30% spent grain inclusion diet; *SG_45* – 45% spent grain inclusion diet; *SG_60* – 60% spent grain inclusion diet; *SG_75* – 75% spent grain inclusion diet). |
| *Bin* | Bin number (1-15). |
| *Hatch_Start_Date_Previous* | Date (YYYY-MM-DD) that the eggs laid by the previous generation started hatching. |
| *Generation_Start_Date* | Date (YYYY-MM-DD) that the hatchlings were added to their respective bins to begin the generation. |
| *First_Adults_Observed_Date* | Date (YYYY-MM-DD) that the first adults were observed in a bin. |
| *First_Adults_Observed_Day* | Day (of the generation) that the first adults were observed in a bin. |
| *Egg_Laying_Start_Date* | Date (YYYY-MM-DD) that the egg laying substrate was provided to a bin. |
| *Egg_Laying_End_Date* | Date (YYYY-MM-DD) that the egg laying substrate was removed from the bin. |
| *Harvest_Date* | Date (YYYY-MM-DD) that the adult crickets were harvested. |
| *Hatch_Start_Date* | Date (YYYY-MM-DD) that the eggs from that respective generation began to hatch. |
