# Supplementary material for: Farmed cricket performance remains stable over five generations of rearing on a waste-based diet
Source: J Econ Entomol. 2026 Apr 16;119(3):1688–98. doi: 10.1093/jee/toag089 (PMC13268526; doi:10.1093/jee/toag089)
Supplement: toag089_Supplementary_Data [file toag089_supplementary_data.zip › toag089_Supplementary_Data/Supplemental_Tables_and_Figures.docx]

**Farmed cricket performance remains stable over five generations of rearing on a waste-based diet**

Kasdorf, S.Y., Bertram, S.M. and MacMillan, H.A.

**Supplemental Tables & Figures**

**Figure S1.** Total volume of feed in fluid ounces provided to crickets per generation on a control feed (standard farm feed, turquoise), a 75% spent grain feed (pink) or a feed that increased incrementally from 15-75% spent grain with each generation (purple) to maintain *ab libitum* feeding.

**Figure S2.** Average daily temperature (°C) of the greenhouse used to house crickets during the multigenerational experiment. The data shown is from 2023-12-01 (YYYY-MM-DD) to 2024-05-15 (YYYY-MM-DD). The experiment ran from 2023-12-01 (YYYY-MM-DD) to 2024-08-11 (YYYY-MM-DD). A drop in average daily temperature is visible for a period of time at the end of March.

**Table S1**. Linear Mixed Model Summary for: log(Mass) ~ Diet * Gen_centered * Sex + (1 | Bin)

| Predictor | Estimate | SE | df | t | p-value |
| --- | --- | --- | --- | --- | --- |
| (Intercept) | -1.138 | 0.028 | 114.878 | -40.300 | < 0.001 |
| DietSG_75 | -0.265 | 0.040 | 114.878 | -6.633 | < 0.001 |
| DietSG_V | 0.094 | 0.040 | 114.878 | 2.349 | 0.021 |
| Gen_centered | 0.005 | 0.010 | 726.000 | 0.460 | 0.646 |
| SexM | -0.455 | 0.036 | 726.000 | -12.535 | < 0.001 |
| DietSG_75:Gen_centered | 0.016 | 0.015 | 726.000 | 1.094 | 0.274 |
| DietSG_V:Gen_centered | -0.078 | 0.015 | 726.000 | -5.270 | < 0.001 |
| DietSG_75:SexM | 0.130 | 0.051 | 726.000 | 2.540 | 0.011 |
| DietSG_V:SexM | 0.040 | 0.051 | 726.000 | 0.783 | 0.434 |
| Gen_centered:SexM | 0.008 | 0.015 | 726.000 | 0.548 | 0.584 |
| DietSG_75:Gen_centered:SexM | -0.030 | 0.021 | 726.000 | -1.432 | 0.153 |
| DietSG_V:Gen_centered:SexM | 0.001 | 0.021 | 726.000 | 0.057 | 0.954 |
| SD (Bin) | 0.026 |  |  |  |  |
| Residual SD | 0.166 |  |  |  |  |

Model R² (%): Marginal = 64.5, Conditional = 65.3.

**Table S2**. Linear Mixed Model Summary for: PC1 ~ Diet * Gen_centered * Sex + (1 | Bin)

| Predictor | Estimate | SE | df | t | p-value |
| --- | --- | --- | --- | --- | --- |
| (Intercept) | 1.687 | 0.158 | 737 | 10.700 | < 0.001 |
| DietSG_75 | -0.646 | 0.223 | 737 | -2.897 | 0.004 |
| DietSG_V | 0.179 | 0.223 | 737 | 0.802 | 0.423 |
| Gen_centered | -0.107 | 0.064 | 737 | -1.667 | 0.096 |
| SexM | -2.688 | 0.223 | 737 | -12.053 | < 0.001 |
| DietSG_75:Gen_centered | 0.058 | 0.091 | 737 | 0.637 | 0.524 |
| DietSG_V:Gen_centered | -0.117 | 0.091 | 737 | -1.288 | 0.198 |
| DietSG_75:SexM | 0.165 | 0.316 | 737 | 0.522 | 0.602 |
| DietSG_V:SexM | 0.658 | 0.315 | 737 | 2.086 | 0.037 |
| Gen_centered:SexM | 0.011 | 0.091 | 737 | 0.122 | 0.903 |
| DietSG_75:Gen_centered:SexM | -0.042 | 0.129 | 737 | -0.323 | 0.747 |
| DietSG_V:Gen_centered:SexM | -0.199 | 0.129 | 737 | -1.547 | 0.122 |
| SD (Bin) | 0.000 |  |  |  |  |
| Residual SD | 1.018 |  |  |  |  |

Model R² (%): Marginal = 63.2, Conditional = NA.

Table S3. Generalized Linear Mixed Model Summary for: cbind(Survived, Died) ~ Diet * Gen_centered + (1 | Bin)

| Predictor | Estimate | SE | z value | p-value |
| --- | --- | --- | --- | --- |
| (Intercept) | 1.104 | 0.051 | 21.784 | < 0.001 |
| Diet1 | -0.681 | 0.069 | -9.912 | < 0.001 |
| Diet2 | -0.135 | 0.071 | -1.910 | 0.056 |
| Gen_centered | -0.066 | 0.016 | -4.194 | < 0.001 |
| Diet1:Gen_centered | 0.182 | 0.021 | 8.548 | < 0.001 |
| Diet2:Gen_centered | 0.063 | 0.022 | 2.893 | 0.004 |
| SD (Bin) | 0.120 |  |  |  |

Model R² (%): Marginal = 79.9, Conditional = 87.7.

Table S4. Linear Mixed Model Summary for: First_Adults_Observed_Day ~ Diet * Gen_centered + (1 | Bin)

| Predictor | Estimate | SE | df | t | p-value |
| --- | --- | --- | --- | --- | --- |
| (Intercept) | 29.560 | 0.564 | 69 | 52.375 | < 0.001 |
| DietSG_75 | 1.000 | 0.798 | 69 | 1.253 | 0.214 |
| DietSG_V | -0.600 | 0.798 | 69 | -0.752 | 0.455 |
| Gen_centered | -0.540 | 0.230 | 69 | -2.344 | 0.022 |
| DietSG_75:Gen_centered | -0.580 | 0.326 | 69 | -1.780 | 0.079 |
| DietSG_V:Gen_centered | 0.160 | 0.326 | 69 | 0.491 | 0.625 |
| SD (Bin) | 0.000 |  |  |  |  |
| Residual SD | 1.629 |  |  |  |  |

Model R² (%): Marginal = 30.3, Conditional = NA.

Table S5. Linear Mixed Model Summary for: yield ~ Diet * Gen_centered + (1 | Bin)

| Predictor | Estimate | SE | df | t | p-value |
| --- | --- | --- | --- | --- | --- |
| (Intercept) | 24.814 | 1.008 | 54.048 | 24.605 | < 0.001 |
| DietSG_75 | -2.234 | 1.426 | 54.048 | -1.566 | 0.123 |
| DietSG_V | 10.521 | 1.426 | 54.048 | 7.377 | < 0.001 |
| Gen_centered | 0.329 | 0.399 | 57.000 | 0.823 | 0.414 |
| DietSG_75:Gen_centered | -0.670 | 0.565 | 57.000 | -1.187 | 0.24 |
| DietSG_V:Gen_centered | -4.073 | 0.565 | 57.000 | -7.215 | < 0.001 |
| SD (Bin) | 0.552 |  |  |  |  |
| Residual SD | 2.823 |  |  |  |  |

Model R² (%): Marginal = 65.5, Conditional = 66.

Table S6. Linear Mixed Model Summary for: log(Mass_mg) ~ Diet * Gen_centered + (1 | Bin)

| Predictor | Estimate | SE | df | t | p-value |
| --- | --- | --- | --- | --- | --- |
| (Intercept) | -0.033 | 0.025 | 744 | -1.319 | 0.188 |
| DietSG_75 | -0.081 | 0.035 | 744 | -2.293 | 0.022 |
| DietSG_V | -0.048 | 0.035 | 744 | -1.368 | 0.172 |
| Gen_centered | -0.071 | 0.010 | 744 | -6.942 | < 0.001 |
| DietSG_75:Gen_centered | 0.059 | 0.014 | 744 | 4.106 | < 0.001 |
| DietSG_V:Gen_centered | 0.038 | 0.014 | 744 | 2.636 | 0.009 |
| SD (Bin) | 0.000 |  |  |  |  |
| Residual SD | 0.227 |  |  |  |  |

Model R² (%): Marginal = 7.8, Conditional = NA.

Table S7. Linear Mixed Model Summary for: Hatchling_Yield ~ Diet * Gen_centered + (1 | Bin)

| Predictor | Estimate | SE | df | t | p-value |
| --- | --- | --- | --- | --- | --- |
| (Intercept) | 4.472 | 0.409 | 69 | 10.940 | < 0.001 |
| DietSG_75 | -1.848 | 0.578 | 69 | -3.197 | 0.002 |
| DietSG_V | 0.591 | 0.578 | 69 | 1.023 | 0.31 |
| Gen_centered | -0.727 | 0.167 | 69 | -4.357 | < 0.001 |
| DietSG_75:Gen_centered | 0.512 | 0.236 | 69 | 2.169 | 0.034 |
| DietSG_V:Gen_centered | -0.176 | 0.236 | 69 | -0.746 | 0.458 |
| SD (Bin) | 0.000 |  |  |  |  |
| Residual SD | 1.180 |  |  |  |  |

Model R² (%): Marginal = 45.2, Conditional = NA.
